# Aphtose buccale récidivante, et si c'était une maladie coeliaque

**Published:** 2012-07-27

**Authors:** Kaoutar Zinelabidine, Idrissi Rhizlane, Meriame Meziane, Fatima Zahra Mernissi

**Affiliations:** 1 Service Dermatologie, centre hospitalo-universitaire Hassan II, Fès, Maroc

**Keywords:** Aphtose buccale, maladie cœliaque, récidive, Aphthous ulcer, celiac disease, recurrence

## Abstract

L'aphtose buccale récidivante est une maladie le plus souvent bénigne mais parfois invalidante qui évolue de façon chronique et récidivante. Elle peut être isolée ou associé à d'autre pathologie, d'où l'intérêt de la recherche d'une maladie sous jacente. Nous rapportons le cas d'une aphtose buccale récidivante révélant une maladie coeliaque chez une patiente âgée de 45 ans.

## Introduction

L'aphtose buccale récidivante est une maladie le plus souvent bénigne mais parfois invalidante qui évolue de façon chronique et récidivante. Elle peut être isolée ou associé à d'autre pathologie, d'où l'intérêt de la recherche d'une maladie sous jacente. Nous rapportons le cas d'une aphtose buccale récidivante révélant une maladie coeliaque.

## Patient et observation

**Figure 1:**
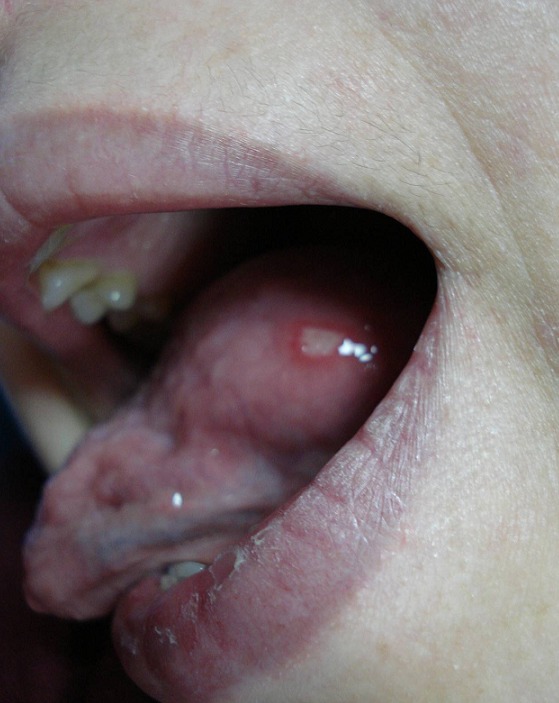
Aphte buccal au niveau la langue

**Figure 2:**
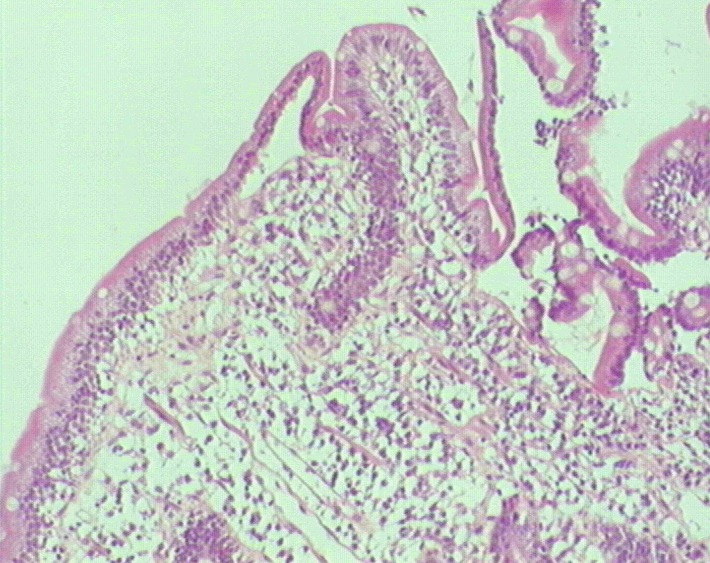
Aplatissement total de la muqueuse jéjunale

**Figure 3:**
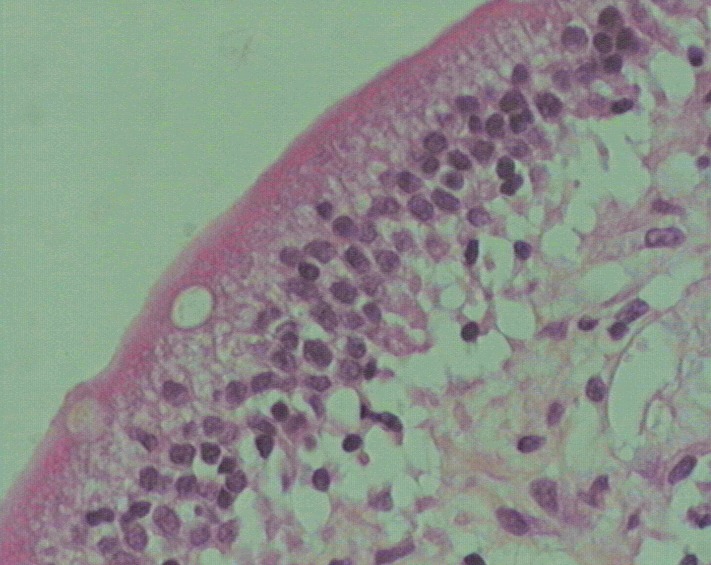
Exocytose lymphocytaire intra-épithéliale > 50 lymphocytes/100 entérocytes

Mme H.F âgée de 45ans, mère de 4 enfants, présentait une aphtose buccale récidivante depuis 14ans, isolée, résistante aux traitements symptomatiques et à la colchicine. L'interrogatoire ne trouvait pas la notion de prise médicamenteuse, de signes digestifs ou d'altération de l'état général. L'examen clinique de la muqueuse buccale avait trouvé des érosions buccales douloureuses ayant un fond beurre frais, entourées d'un halo inflammatoire, siégeant sur le bord latéral de la langue et la face interne de la joue droite (Figure 1). La muqueuse génitale était normale et le reste de l'examen sans particularités. Un bilan biologique a été réalisé objectivant une anémie normochrome normocytaire (Hb=10,2g/d) arégénérative, avec un taux de réticulocytes à 29 000/mm^3^ (20000 - 120000/mm^3^), fer sérique: 0,50mg/l (0,50-1,68mg/l), ferritinémie: 4,7 ng/ml (11-306 ng/ml). Dans le cadre de son anémie, une fibroscopie oeso-gastro-duodénale a été réalisée et qui était normale macroscopiquement, la biopsie jéjunale avait révélé des villosités intestinales de taille raccourcie (Figure 2) avec une exocytose lymphocytaire (> 50 lymphocytes intra épithéliaux /100 entérocytes) (Figure 3). Le dosage des anticorps anti-gliadine, anti-endomysium et anti-transglutaminase était négatif. La patiente a été mise sous régime sans gluten, en association avec un traitement martial pendant 3 mois, avec une bonne évolution. La patiente ne présentait plus d'aphte buccal depuis 18 mois.

## Discussion

Les aphtes récurrents (AR) représentent une affection très fréquente, ils surviennent chez les hommes et les femmes de tous âges, toutes races et toutes régions géographiques. Il a été estimé que 20% de la population générale souffrent d'une aphtose buccale à un certain moment dans leur vie [1] avec des fréquences variables en fonction des pays. Ces différences peuvent être expliquées par les facteurs de l′environnement, de la diététique et surtout des facteurs génétiques [2]. L'aphte se présente sous forme d'une ulcération douloureuse avec un fond jaunâtre ou grisâtre, avec une base infiltrée, des bords nets cerclés par un halo érythémateux. Il est douloureux et sans adénopathie satellite. Sous sa forme sporadique ou récurrente, l'ulcère aphteux est une lésion multifactorielle, favorisé ou déclenché par des facteurs alimentaires, psychiques, traumatiques ou par les périodes menstruelles, les infections et des facteurs toxiques.

La maladie coeliaque (MC) est caractérisée par des changements inflammatoires de la muqueuse de l′intestin grêle induits par un composant de la protéine du gluten [1]. Les forme atypiques sont de plus en plus fréquentes, le diagnostic de la maladie coeliaque doit être évoqué, même en l'absence de symptômes digestifs, devant une anémie ferriprive, une hyposidérémie isolée, une aphtose buccale récidivante, une aménorrhée, une stérilité, des signes neurologiques, une ostéoporose, des arthralgies, une dermatite herpétiforme ou une augmentation des transaminases [3]. Un aspect endoscopique normal est observé dans 1/3 des cas, comme c'était le cas pour notre patiente, et ne doit pas récuser la pratique de biopsie. Cette dernière montre une atrophie villositaire totale ou subtotale, une augmentation des lymphocytes intra-épithéliaux, une hyperplasie des cryptes. La confirmation biologique fait appel aux anticorps anti-endomysium et anti gliadine, une recherche négative n'élimine pas la maladie coeliaque, puisque 5 à 10% des coeliaques ont une sérologie négative [4]. De récentes publications scientifiques mettent en relation les inflammations intestinales avec l'aphtose buccale. Cette relation est maintenant établie, vu l'étroite relation entre le fonctionnement de la muqueuse intestinale et celui de la muqueuse buccale qui partagent la même origine embryologique [5]. La prévalence des AR est plus élevée au cours de la MC (22,7% versus 7,1% chez la population générale) [2]. Cette association a été observée surtout avec les groupes génétiques HLA-DRw10 et DQw1 [1]. Cependant dans l'étude de Sedghizadeh, les auteurs ont conclu que l'association entre les deux affections n'est pas significative et que l'AR doit être considéré, comme un «indicateur de risque " pour la MC plutôt qu′un "facteur de risque" [6]. En effet l'aphtose buccal était le signe révélateur de la MC dans notre cas.

Le régime sans gluten (RSG), bien que peu attractif sur le plan goût et contraignant sur le plan social, constitue un test diagnostic et un traitement efficace de la MC [7, 8]. Les aphtes disparaissent habituellement sous RSG [1, 9]; ainsi dans la série de Campisi, les patients ayant respecté le RSG ont eu une amélioration significative 1 an après le début du RSG, alors qu′aucune amélioration n'a été observée chez les patients qui n'ont pas respecté ce régime [2]. En témoigne l'évolution de notre patiente.

## Conclusion

Le profil clinique de la maladie coeliaque de l'adulte a changé au cours de la dernière décennie, avec la mise en évidence d'une prévalence élevée liée à l'existence de formes cliniques frustes avec des manifestations atypiques non digestives. De ce fait, il faut y penser devant une aphtose buccale récidivante.

## References

[1] Natah SS, Konttinen YT, Enattah NS (2004). Recurrent aphthous ulcers today: a review of the growing knowledge. Int J Oral Maxillofac Surg.

[2] Campisi G, Di Liberto C, Carroccio A, Compilato D, Iacono G (2008). Coeliac disease: oral ulcer prevalence, assessment of risk and association with gluten-free diet in children. Dig Liver Dis.

[3] Cellier C, Grosdidier E (2001). Maladie coeliaque de l'adulte. La revue du praticien.

[4] Cosnes J (2009). Maladie coeliaque et régime sans gluten. Gastroentérologie Clinique et Biologique.

[5] Aydemir S, Tekin NS, Aktunç E (2004). Celiac disease in patients having recurrent aphthous stomatitis. Turk J Gastroenterol.

[6] Sedghizadeh PP, Shuler CF, Allen CM, Beck FM, Kalmar JR (2002). Celiac disease and recurrent aphthous stomatitis: a report and review of the literature. Oral Surg Oral Med Oral Pathol Oral Radiol Endod.

[7] Olives JP (2006). Maladie cœliaque: nouvelles perspectives. Médecine thérapeutique/Pédiatrie.

[8] Tkoub EM (2008). Maladie coeliaque de l'adulte. Revue française d'allergologie et d'immunologie clinique.

[9] Zone JJ (2005). Skin Manifestations of Celiac Disease. Gastroenterology.

